# Development and Utility of an Internal Threshold Control (ITC) Real-Time PCR Assay for Exogenous DNA Detection

**DOI:** 10.1371/journal.pone.0036461

**Published:** 2012-05-03

**Authors:** Weiyi Ni, Caroline Le Guiner, Philippe Moullier, Richard O. Snyder

**Affiliations:** 1 Department of Molecular Genetics and Microbiology, College of Medicine, University of Florida, Gainesville, Florida, United States of America; 2 Laboratoire de Thérapie Génique, INSERM UMR1089, IRT UN, Nantes, France; 3 Center of Excellence for Regenerative Health Biotechnology, University of Florida, Alachua, Florida, United States of America; University of Michigan School of Medicine, United States of America

## Abstract

Sensitive and specific tests for detecting exogenous DNA molecules are useful for infectious disease diagnosis, gene therapy clinical trial safety, and gene doping surveillance. Taqman real-time PCR using specific sequence probes provides an effective approach to accurately and quantitatively detect exogenous DNA. However, one of the major challenges in these analyses is to eliminate false positive signals caused by either non-targeted exogenous or endogenous DNA sequences, or false negative signals caused by impurities that inhibit PCR. Although multiplex Taqman PCR assays have been applied to address these problems by adding extra primer-probe sets targeted to endogenous DNA sequences, the differences between targets can lead to different detection efficiencies. To avoid these complications, a Taqman PCR-based approach that incorporates an internal threshold control (ITC) has been developed. In this single reaction format, the target sequence and ITC template are co-amplified by the same primers, but are detected by different probes each with a unique fluorescent dye. Sample DNA, a prescribed number of ITC template molecules set near the limit of sensitivity, a single pair of primers, target probe and ITC probe are added to one reaction. Fluorescence emission signals are obtained simultaneously to determine the cycle thresholds (Ct) for amplification of the target and ITC sequences. The comparison of the target Ct with the ITC Ct indicates if a sample is a true positive for the target (i.e. Ct less than or equal to the ITC Ct) or negative (i.e. Ct greater than the ITC Ct). The utility of this approach was demonstrated in a nonhuman primate model of rAAV vector mediated gene doping *in vivo* and in human genomic DNA spiked with plasmid DNA.

## Introduction

The availability of simplified and controlled tests for detecting exogenous DNA molecules is of great demand in infectious disease diagnosis, evaluating biodistribution of vectors used in legitimate gene therapy clinical trials, and detection of illicit gene doping. Real-time PCR can detect specific DNA signals, even at a very low concentration with reliability and specificity. In addition, real-time PCR is amenable to automation and remote data collection for high throughput screening. Taqman real-time PCR, using specific sequence probes, provides an efficient method to detect exogenous DNA accurately and quantitatively. However, to determine with confidence if the PCR signal is not a false positive or a false negative requires multiple controls. In addition, for quantitative determination of copy number, external copy number standard curves are required and pose a risk of contaminating the laboratory.

Several viral-based and non-viral based gene transfer systems are being developed and evaluated in human clinical trials. These systems have demonstrated the ability to safely and efficiently deliver therapeutic transgenes to a variety of tissues of animals and humans, and examples of therapeutic benefit in humans are increasing [1], [2], [3], [4], [5], [6], [7]. Recombinant adeno-associated viral (rAAV) vectors and naked plasmid are two such gene transfer systems capable of gene delivery to skeletal muscle of animals [8], [9], [10], [11], [12] and humans [13], [14], [15], [16]. Recently, as an alternative to direct injection, regional vascular infusion of vector to achieve skeletal muscle transduction has been reported for plasmid DNA [17], [18] and for rAAV vectors [19], [20].

When developing legitimate gene transfer modalities for gene therapy and vaccination, the evaluation of the distribution of vector sequences in pre-clinical animal studies and human clinical trials is required by regulatory agencies to determine the level of gene delivery to the target tissue and non-target tissues transduced collaterally, and to evaluate vector shedding into the environment. The type of vector, the delivery method, injection schedule, route of administration, and administrated dose are the main variables to impact biodistribution and shedding [20], [21], [22], [23], [24]. To date, traditional real-time PCR assays have been used to analyze rAAV vector distribution after administration in human trials [25] or animal models [9] [26] [27], [28], [29]. To control for the presence of different potential inhibitors for each tissue source (eg. matrix effects), a common practice is to analyze each sample in duplicate with one of the duplicates spiked with a known number of copies of a plasmid harboring the PCR target sequences.

Sports organizations have increased their focus on developing efficient ways to curtail the illegitimate use of genes or genetic elements that have the capacity to enhance athletic performance, also referred to as gene doping [30]. These organizations recognize that gene doping has the potential to threaten the integrity of sport, undermine principles of fair play in sport, imposes potential harm to non-doping athletes, and involves major health risks to athletes [31]. Our previous study demonstrated that rAAV viral vectors can be detected by standard real-time PCR assays in macaque white blood cell genomic DNA (gDNA) up to 57 weeks after intramuscular injection. The sensitivity of these assays was validated to 3 copies in the presence of 500 ng macaque gDNA [32].

One of the major challenges in standard real-time PCR analysis is how to eliminate the false negative signals which can be caused by inhibitors [33] or inefficient PCR conditions. Although multiplex Taqman PCR assays have been applied in an effort to address problems of reliability, such as by adding an extra primer-probe sets targeted to other endogenous DNA sequences (housekeeping genes), however, the sequence and secondary structure differences between primers and probe binding sites, and amplified sequences contribute to different detection efficiencies. Competitive PCR methods can be used to quantify DNA copy number, however, the method is limited by the necessity of assembling multiple competitive reactions for a single determination and, most notably, the need for a post-PCR electrophoresis-based detection and analysis step. In realizing these problems and difficulties of utilizing real-time PCR to detect exogenous DNA sequences, different Taqman PCR assays which use internal controls have been applied in an effort to address problems of reliability, such as by adding an extra primer-probe set targeted to other endogenous DNA sequences [34] or exogenous targets [34], [35], [36], [37], [38], [39], [40], [41]. These previous approaches were primarily aimed at controlling sample adequacy, eliminating false negative results, performed in separate reactions, did not share the same primers, or preferentially amplified the target.

Here, we describe a simplified real-time PCR format that controls for false negative results and establishes true positive results, since the copy number of a positive sample must equal or surpass the copy number of a synthetic internal control. This system includes the use of a synthetic internal threshold control (ITC) template and corresponding ITC probe. Sample DNA, a prescribed number of ITC template molecules, a single pair of primers, target probe, and ITC probe are added to one reaction. Fluorescence emission signals are obtained simultaneously to determine the cycle thresholds (Ct) for amplification of the target and ITC sequences. Comparison of the Ct from the ITC and the Ct of the target is the parameter used to determine if a test sample is positive or negative for the presence of a homologous or nonhomologous exogenous DNA sequence.

## Materials and Methods

### Recombinant human and macaque EPO plasmid

The pShuttle-CAG-hEPO-pA plasmid contains the human erythropoietin (hEPO) cDNA under the control of the CAG promoter and the SV40 polyA (pA) sequence. The pSSV9-MD2-cmEPO rAAV vector plasmid harbors the cmEPO transgene under the control of the CMV promoter and SV40 pA. The integrity of the plasmids was verified by complete sequencing.

### Ethics Statement

Human whole blood samples were obtained from healthy donors following institutional-approved protocols from the Etablissement Français du Sang, Nantes, France.

Macaque whole blood samples were obtained from the same animals used in the studies previously described by Ni et al [32]. Experiments were conducted on 3–5 kg captive-bred cynomolgus macaques purchased from BioPrim (Baziege, France). Macaques were housed in an enriched environment (toys, fresh fruits and vegetables) and were monitored daily for health and welfare. The Institutional Animal Care and Use Committee of the Pays de Loire (France) approved the protocol (permit number #2007.6). The research was conducted at the Boisbonne Centre (ONIRIS, Nantes) under the authorization #5937C delivered by the Departmental Direction of Veterinary Services (Loire-Atlantique, France) and in accordance with the recommendations of the Weatherall report: “The use of nonhuman primates in research".

In order to avoid any discomfort during and after the experiments, all procedures were carried out after animal sedation with 30 µg/kg of Medetomidine (DomitorH, Pfizer) and 7 mg/kg of Ketamine (ImalgeneH, Merial). Intramuscular injections of rAAV vectors and blood samplings were classified as mild severity procedures. Special attention was paid to the health and welfare of animals during the work, and blood samples were collected regularly to follow biochemical and hematological parameters. In particular, creatine phosphokinase levels were monitored in the 4 macaques and all stayed in the normal range before injection and throughout the protocol after administration of the rAAV vector.

### rAAV vector production and administration to macaque skeletal muscle

The rAAV vector production and its administration to macaque skeletal muscle was described previously [32]. Briefly, for direct rAAV IM injections, the total dose was split over one or two pre-tattooed injection sites along the Tibialis Anterior muscle in a maximal volume of 600 µl. Mac 3 was injected with 2.5E10 vg/kg of the rAAV1-MD2-cmEPO vector. Mac 4 was injected with 2.5E11 vg/kg of the rAAV1-MD2-cmEPO vector. Mac 5 was injected with 5E9 vg/kg of the rAAV8-MD2-cmEPO vector. Mac 6 was injected with 2.5E10 vg/kg of the rAAV8-MD2-cmEPO vector.

### DNA extraction from human and macaque white blood cells

Human (naïve) and macaque (naïve and rAAV transduced) whole blood was collected and DNA extracted from the white blood cell (WBC) pellet using the Gentra Puregene kit (cat # 158467) from Qiagen, as previously described [32]. Concentration and purity of the gDNA was determined using a nano-spectrophotometer from Implen. Integrity of the DNA was verified by migration of 3 µg of total DNA on a 0.8% agarose gel, followed by Ethidium Bromide staining, and for macaque DNA by real-time PCR of the endogenous macaque ε-globin gene as previously described [32].

### ITC assay Development: Primer-Probe design

Primers and probes were designed using ABI Primer Express 3.0 based on the cytomegalovirus (CMV) immediate early promoter in the pSSV9-MD2-cmEPO plasmid, the human EPO gene (NC_000007.13), or macaque EPO gene (NC_007860.1). Because of the constraints of the EPO Exon-Exon junction sequences and the required Tm, screening of multiple primer-probes combinations was required. Primer and probe sequences were screened *in silico* against the human genome and the macaque genome (http://genome.ucsc.edu/cgi-bin/hgPcr?command=start). Primers, probes and ITC templates were quantified using a nano- spectrophotometer from Implen.

#### a. ITC^cmEPO^ duplex assay

The cmEPO primer-probe assay targets the cmEPO Exon2–3 junction, harbored in pSSV9-MD2-cmEPO plasmid. The ITC^cmEPO^ duplex assay was designed with forward primer 5′AATGAGAATATCACCGTCCCAGAC3′, reverse primer 5′AGCTTCTGAGAGCAGGGCC3′, cmEPO probe 6FAM-AAGAGGATGGAGG TCGG-MGBNFQ and ITC probe 6VIC-CGGCCATTTTCCA-MGBNFQ. The ITC probe targets ITC^cmEPO^ template of two complimentary synthetic single strand DNAs that were annealed. The sequence of the forward ITC template sequence is 5′GAATGAGAATATCACCGTCCCAGACACCAAAGTTAACTT CTATGCCTGGAAGACGGCCATTTTCCAAGCAGGCTGTAGAAGTCTGGCAGGGCCTGGCCCTGCTCTCAGAAGCTGACGT3′ and the sequence of the complementary sequence is 5′CAGCTTCTGAGAGCAGGGCCAGGCCCTGCCAGACTTCTACA GCCTGCTTGGAAAATGGCCGTCTTCCAGGCATAGAAGTTAACTTTGGTGTCTGGGACGGTGATATTCTCATTCTGCA.3′.

#### b. ITC^hEPO^ duplex assay

The hEPO primer-probe assay targets the hEPO Exon3–4 junction, harbored in pShuttle-CAG-hEPO-pA plasmid. The ITC^hEPO^ duplex assay was designed with forward primer 5′TGAATGAGAATATCACTGTCCCAGAC3′, reverse primer 5′CTTCCGACAGCAGGGCC3′, hEPO probe 6FAM-AAGAGGATG GAGGTCGG-MGBNFQ and ITC probe VIC-CGGCC ATTTTCCA-MGBNFQ. The ITC probe targets ITC^hEPO^ template of two complementary synthetic single strand DNAs that were annealed. The sequence of the forward ITC template is 5′GTGAATGAGAATATCACTGTCCCAGACACCAAAGTTAACTTC TATGCCTGGAAGACGGCCATTTTCCAAGCAGGCTGTAGAAGTCTGGCAGGGCCTGGCCCTGCTGTC GGAAGGACGT3′ and the sequence of the complementary ITC sequence is 5′CCTTCCGACAGCAGGGCCAGGCCCTGCCAGACTTCTACAGC CTGCTTGGAAAATGGCCGTCTTCCAGGCATAGAAGTTAACTTTGGTGTCTGGGACAGTGATATTCTC ATTCACTGCA3′.

#### c. ITC^CMV^ duplex assay

The CMV primer-probe assay targets the CMV immediate early promoter junction, harbored in pSSV9-MD2-cmEPO plasmid. The ITC^CMV^ duplex assay was designed with forward primer 5′AATGGGCGGTAGGCGTGTA3′, reverse primer 5′CGATCTGACGGTTCACTAAACG3′, CMV probe 6FAM-TGGGAGGT CTATATAAGC–MGBNFQ and ITC probe VIC-CG GCCATTTTCCA-MGBNFQ. The CMV ITC probe targets ITC^CMV^ template of two complementary synthesized single strand DNAs that were annealed. The sequence of the forward ITC template is 5′CCGATCTGACGGTTCACTAAACGAGCTCTTGGAAAATGGCCGCCGTAC ACGCCTACCGCCCATTCTGCA3′ and the sequence of the complementary sequence is 5′GAATGGGCGGTAGGCGTGTACGGCGGCCATTTTCCAAGAGCTCGTTTAG TGAA CCGTCAGATCGGACGT3′.

### Traditional real-time PCR program

Taqman Real-time PCR conditions were optimized with primers and their corresponding fluorescent probes. The concentrations of 250 nM probe and 900 nM primers were found to be optimal. 500 ng of each DNA sample was amplified in a final volume of 25 µL containing 1× TaqMan® Universal PCR Master Mix (Applied Biosystems cat# 4304437). Amplification was performed using an ABI StepOnePlus PCR machine with an initial incubation at 50°C for 2 min, a denaturation at 95°C for 10 min, then 40 cycles of denaturation at 94°C for 15 s and an annealing/extension step at 60°C for 1 min. During thermal cycling, emission from each sample was recorded and ABI StepOne software v2.0 processed the raw fluorescence data to produce threshold cycle (Ct) values for each sample.

### ITC duplex assay real-time PCR program

500 ng of macaque gDNA sample (for ITC^cmEPO^ duplex assay) or human gDNA (for ITC^hEPO^ and ITC^CMV^ duplex assays) was amplified in a final volume of 30 µL containing 1× TaqMan® Universal PCR Master Mix (Applied Biosystems cat# 4304437) or 1× TaqMan® Fast Virus 1-Step Master Mix with reverse transcriptase (Applied Biosystems cat# 4444432). 5 copies or 10 copies of the corresponding ITC template were added in each reaction system. DMSO (Sigma D2650) was added in the final master mix to increase the assay sensitivity. Amplification was performed using an ABI StepOnePlus PCR machine with an initial incubation at 50°C for 2 min, a denaturation at 95°C for 10 min, then 40 cycles of denaturation at 94°C for 15 s and an annealing/extension step at 60°C for 1 min. During thermal cycling, 6FAM and VIC fluorescence emissions were recorded and ABI StepOne software v2.0 processed the raw 6FAM fluorescence data to produce threshold cycle (Ct) values for testing samples and VIC fluorescence data for ITC templates.

### Statistical Analyses

#### a. SAS Analyses

SAS 9.2 software was utilized to analyze data from ITC assays. SAS T-Test procedure was applied to compare the Ct values from each ITC assays. SAS GLM procedure was used to perform One-Way ANOVA and Two-Way ANOVA analyses.

#### b. Equivalence Testing

Independent sample equivalence testing was performed [42]. The null hypothesis (H_0_) is Ct_Target_>Ct_ITC_ and Ct_Target_<Ct_ITC_; The alternative hypothesis (H_A_) is Ct_Target_ = Ct_ITC_. The tolerance limit (Δ) was set to 0.5 Ct to evaluate the parity of the Ct's from the two different real-time PCR reactions in the duplex assay. The confidence level (α) was set to 0.10. The critical t-value is t_α, 2*n−2_ which is obtained from the Student's t-test distribution table. The observed t-values are calculated:
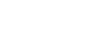


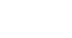

*d* is the difference in the mean Ct between the target sequence and corresponding ITC template. *S_d_* is the pooled standard deviation of the two independent samples:
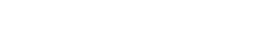



*n*: Number of replicates

*S_1_*: Standard deviation of Ct_target_.

*S_2_*: Standard deviation of Ct_ITC_.

The rejection rule is: if t_ci_ and t_cs_>t _α, 2*n−2_, then reject H_0_.

## Results

A novel real-time PCR-based approach for detecting exogenous DNA sequences has been developed. The system includes the use of an internal threshold control (ITC) template and corresponding ITC probe. The implementation of the ITC involves maintaining the melting temperature (Tm) of the ITC probe similar to the Tm of the probe used for the target, having the distance between the primer hybridization sites on the ITC template similar to the distance in the target, and labeling the ITC probe with a fluorescent dye different than the target probe. In this duplex assay format, the target and ITC template are co-amplified by the same primers, but are detected by two different probes each with a different fluorescent dye. Sample DNA, a prescribed number of ITC template molecules set near the limit of sensitivity, target primers, target probe and ITC probe are amplified in one reaction. Fluorescence emission signals are obtained by the real-time PCR machine simultaneously to determine the cycle thresholds (Ct) for amplification of the target and ITC sequences. Comparison of Ct from the ITC and Ct of the target is the parameter used to determine if test samples are positive or negative for the targeted DNA sequence. For the ITC duplex assays, if the Ct of the target probe is less than or equal to the Ct of the ITC probe, then the sample is considered a true positive (meaning that there is the same or more copies of the sequence of interest relative to the ITC template). Additionally, if the Ct of the Target probe is greater than the Ct of the ITC probe, then the sample is considered negative.

**Figure 1:**
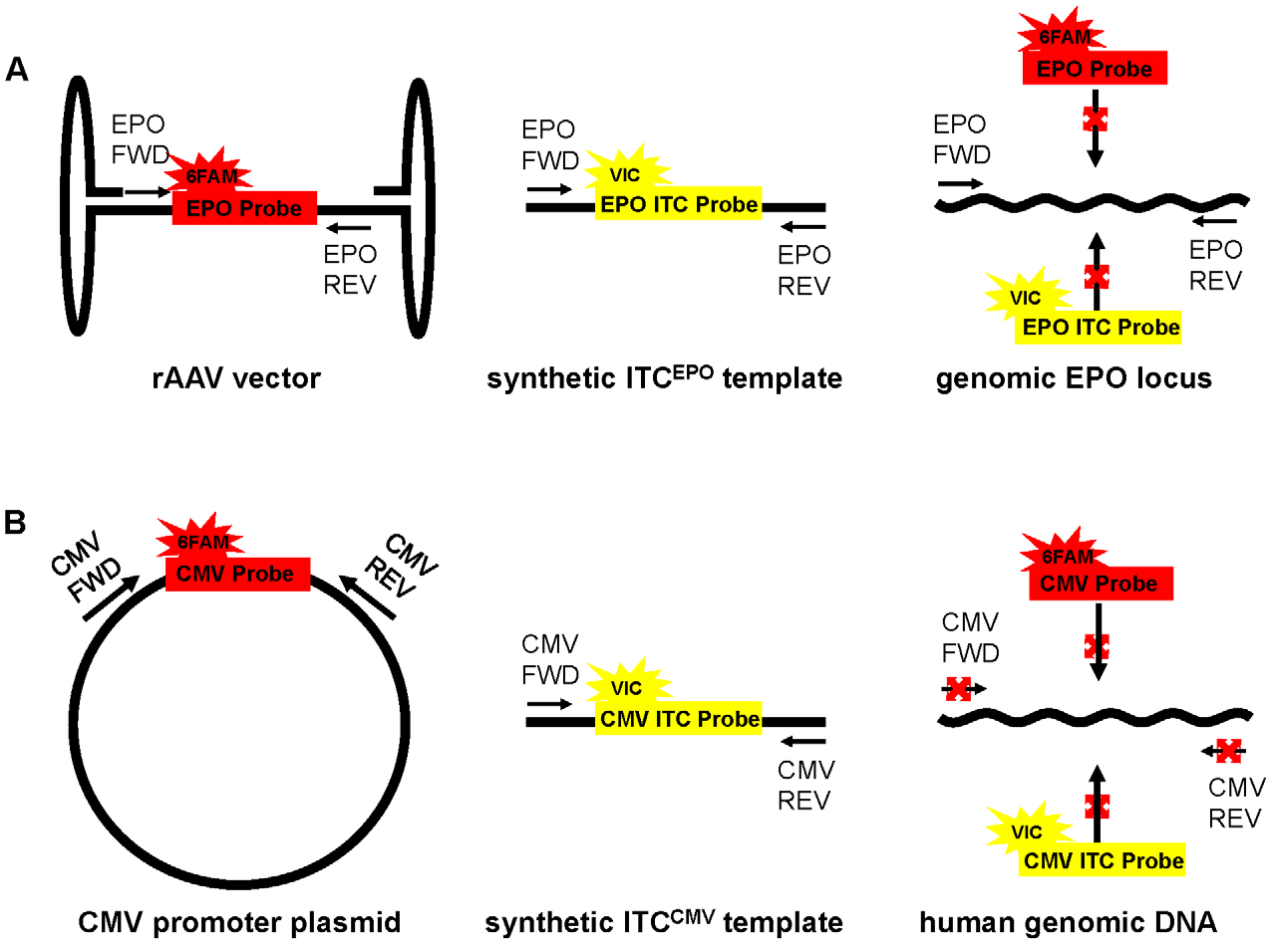
Internal Threshold Control (ITC) assay format. **Panel (A)** ITC^EPO^ duplex assay format. The EPO probe specifically detects the EPO cDNA harbored in rAAV vectors and does not bind to the genomic DNA because it rests on an exon/exon junction. The EPO ITC probe recognizes only the synthetic ITC^EPO^ template. The EPO primers recognize the EPO cDNA, the ITC^EPO^ template, and the genomic EPO locus. **Panel (B)** ITC^CMV^ duplex assay format. The CMV probe specifically detects the CMV immediate early promoter region in the pSSV9-MD2-cmEPO plasmid. The CMV ITC probe recognizes only the synthetic ITC^CMV^ template. The CMV primers recognize the CMV promoter and the ITC^CMV^ template.

**Figure 1** represents the features and rationale of applying the ITC to detect non-endogenous sequences that are either homologous or non-homologous with genomic DNA. Two different ITC^EPO^ duplex assays were developed that target macaque or human EPO cDNA. These ITC^EPO^ duplex assays are performed in the presence of the endogenous homologous genomic locus, thus the ITC assay needs to distinguish the cDNA carried in a gene transfer vector from the cellular host genomic DNA. For this, the EPO primer-probe assays were designed to target an EPO Exon-Exon junction (Exon 2–3 junction for cmEPO and Exon 3–4 junction for hEPO). The synthetic ITC^EPO^ template includes a different probe binding site and maintains the flanking EPO sequences including the EPO primer binding sites. As a consequence, the EPO primers recognize the EPO cDNA in the viral vector, along with the ITC^EPO^ template, and macaque or human gDNA. However, the EPO probe (6FAM dye) specifically detects the EPO cDNA, and the EPO ITC probe (VIC dye) specifically detects the EPO ITC template and neither can detect genomic DNA (**Figure 1A**).

To demonstrate the applicability of the ITC duplex assay approach to address infectious agent detection, we also developed an ITC duplex assay in which the cytomegalovirus (CMV) promoter, used in the pSSV9-MD2-cmEPO plasmid, was targeted. The CMV promoter PCR target developed here is homologous to the promoter found in most CMV strains, including: the Towne strain (Genbank AY315197) that is the basis of the National Institute of Standards and Technology (NIST) Reference plasmid, the Merlin strain (Genbank AY446894) used in the World Helath Organization (WHO) reference standard (National Institute for Biological Standards and Control (NIBSC) product # 09/162), the JP strain (Genbank GQ221975) from a clinical specimen, and cell culture strains HAN38 (Genbank GQ396662) and VR1814 (Genbank GU179289). Since the heterologous CMV sequences are targeted, there is no competition with the host genomic DNA (**Figure 1B**).

Traditional real-time PCR assays to detect the hEPO cDNA and CMV promoter were first developed using an approach similar to our previously published assay for cmEPO cDNA [32]. Design of the corresponding synthetic ITC template for each of these targets requires that (1) the distance between the primers is similar to the distance between the same primer sites on the target; (2) the target probe and ITC probe have similar Tm; (3) the PCR products have similar Tm; (4) there is no homology between the target probe and ITC template nor between ITC probe and target sequences. For each assay the primers and probes were verified *in silico* to ensure that there was no cross hybridization between primers, primers and probes, primers/probe sets and EPO cDNA or genomic DNA or ITC template. The PCR products of each individual PCR assay were analyzed by agarose gel electrophoresis and demonstrated a single band (data not shown). The performance of each individual assay was evaluated.

### Performance and detection limits of individual assays

Traditional real-time PCR was performed to evaluate the lower limit of quantitation and linearity of the six individual assays. A titration of the pSSV9-MD2-cmEpo plasmid in the presence of naïve macaque genomic DNA illustrates that the cmEPO assay is capable of detecting 5 copies. Likewise, a titration of the ITC^cmEPO^ template illustrates that the ITC^cmEPO^ assay is capable of detecting 5 copies in the presence of naïve macaque genomic DNA. The sensitivities of the individual hEPO, ITC^hEPO^, CMV, ITC^CMV^ assays in the presence of naïve human genomic DNA is 10 copies. The linearity of all the six assays is above 0.98 over an 8 log dynamic range from 10 to 1E9 copies (**Table 1**). The reduction in efficiencies seen in the presence of gDNA most likely reflects competition for primers. To evaluate the accuracy of detecting the sequences at the lower limit of quantitation, 15 replicates were performed for cmEPO and ITC^cmEPO^ assays at 5 copies and at 10 copies for the other four assays and the means and standard errors of copy number and Ct were calculated (**Table 2**).

**Table 1:** Individual assay linearity and efficiency.

Assay	Linearity (R^2^)	Linearity (R^2^)	Efficiency (%)	Efficiency (%)
	in the absence of 500 ng gDNA	in the presence of 500 ng gDNA*	in the absence of 500 ng gDNA	in the presence of 500 ng gDNA
**cmEPO**	0.992	0.989	95	82
**ITC^cmEPO^**	0.989	0.998	91	86
**hEPO**	0.999	0.998	92	88
**ITC^hEPO^**	0.996	0.993	89	86
**CMV**	0.996	1.000	99	97
**ITC^CMV^**	0.997	0.989	98	94

* macaque gDNA with cmEPO and ITC^cmEPO^ assays, and human gDNA with hEPO, ITC^hEPO^, CMV and ITC^CMV^ assays.

**Table 2:** Individual assay sensitivity.

Assay	Positive Control	Mean* Copy Number (s.e.)	Mean* Ct (s.e.)	CV (%) of Ct
**cmEPO**	5 copies pDNA with 500 ng gDNA (macaque)	6.7 (2.5)	37.24 (0.22)	2.2
	5 copies pDNA with TE	5.9 (3.4)	37.47 (0.28)	2.9
**ITC** ^cmEPO^	5 copies ITC template with 500 ng gDNA (macaque)	6.5 (2.7)	37.22 (0.24)	1.5
	5 copies ITC template with TE	5.5 (3.8)	37.14 (0.31)	3.2
**hEPO**	10 copies pDNA with 500 ng gDNA (human)	9.6 (3.3)	36.74 (0.14)	1.5
	10 copies pDNA with TE	10.8 (2.8)	36.45 (0.10)	1.1
**ITC^hEPO^**	10 copies ITC template with 500 ng gDNA (human)	11.8 (3.9)	36.55 (0.14)	1.6
	10 copies ITC template with TE	11.1 (2.4)	36.73 (0.10)	0.9
**CMV**	10 copies pDNA with 500 ng gDNA (human)	11.9 (5.3)	36.29 (0.18)	2.0
	10 copies pDNA with TE	11.1 (4.1)	36.35 (0.14)	1.5
**ITC^CMV^**	10 copies ITC template with 500 ng gDNA (human)	10.7 (4.3)	36.64 (0.16)	1.7
	10 copies ITC template with TE	11.8 (4.0)	36.55 (0.13)	1.4

* From fifteen replicates of plasmid (pDNA) or ITC template. Standard error, s.e., (in parenthesis).

### Specificity of the individual assays

The specificity of the individual assays was tested (**Table 3**). A lack of signal at 40 cycles was defined as “negative" and a Ct signal before 40 cycles of amplification was considered “positive". 500 ng of naïve macaque or human gDNA were used as samples. **Table 3** demonstrates that no false positive signals were detected in 20 replicates of each of the six individual assays caused by either non-targeted exogenous or endogenous DNA sequences, or laboratory contamination. To confirm that we are detecting the vector genome harboring the EPO cDNA and not residual endogenous mRNA, we obtained the copy number signal with our standard master mix and this signal was not influenced by RNase. Furthermore, when a master mix that utilizes a reverse transcriptase step was used, endogenous mRNA was detected and this RT-dependent signal was reduced with an RNase A pre-treatment (data not shown).

**Table 3:** Individual assay specificity.

Assay	Negative Control	False positive	False Positive Rate (%)
**cmEPO**	500 ng gDNA (macaque)	0/20	0
**ITC** ^cmEPO^	500 ng gDNA (macaque)	0/20	0
**hEPO**	500 ng gDNA (human)	0/20	0
**ITC^hEPO^**	500 ng gDNA (human)	0/20	0
**CMV**	500 ng gDNA (human)	0/20	0
**ITC^CMV^**	500 ng gDNA (human)	0/20	0

### Equivalence testing of ITC duplex assays

**Figure 2:**
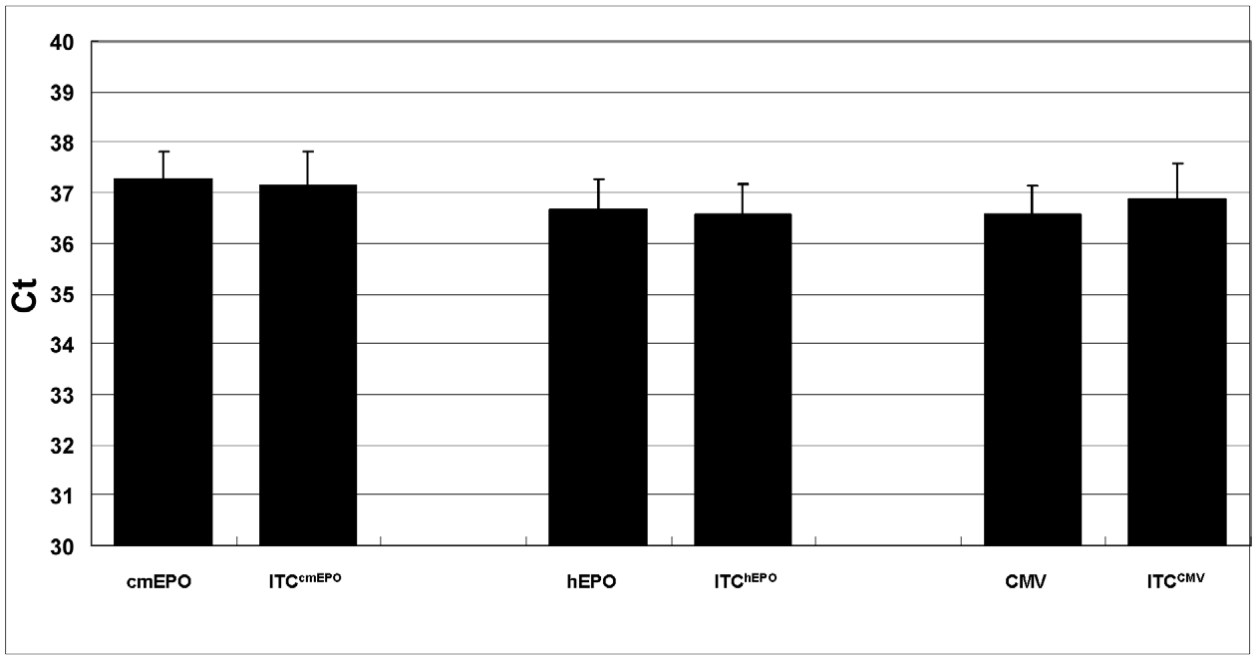
ITC duplex assay equivalence testing. All three ITC duplex assays were tested in the presence of same amount of target and ITC template and 500 ng naïve gDNA. Five copies of pSSV9-MD2-cmEPO plasmid and ITC^cmEPO^ template were amplified using the ITC^cmEPO^ duplex assay. 10 copies of pShuttle-CAG-hEPO-pA plasmid and ITC^hEPO^ template were amplified using the ITC^hEPO^ duplex assay. 10 copies of pSSV9-MD2-cmEPO plasmid and ITC^CMV^ template were amplified using the ITC^CMV^ duplex assay. Each reaction was repeated 15 times. Similarities in mean Ct values were analyzed statistically as described in Materials and Methods.

Each of the three individual target assays was paired with the corresponding ITC assay to create the three ITC duplex assays. The ITC duplex assay format includes the requirement that same copy number of target sequences and corresponding ITC templates give similar Ct's. In cases where the P-value shows a statistically significant difference, such as when the Ct of the target is significantly higher or lower than the Ct of the ITC, then the Student's t-test can be used. However, when the Ct of the target is equal to the Ct of the ITC, the equivalency needs to be confirmed statistically to be able to designate the sample as a true positive. Equivalence testing of our three ITC duplex assays was performed. Each assay was used to detect target DNA sequences near their limit of sensitivity in the presence of an equal amount of ITC template and 500 ng of naïve gDNA (**Figure 2**). Fifteen replicates were performed and the Ct's were recorded and analyzed. First, large P-values (cmEPO P = 0.5816, hEPO P = 0.6783, CMV P = 0.4819) were obtained by the Student's t-test suggesting that no significant differences exist between each target Ct and the corresponding ITC Ct from all three duplex assays. To determine if the Ct values were statistically equivalent, equivalence testing was conducted (see Materials and Methods), where the tolerance limit was set as 0.5 Ct (equal to 1.5% of the max Ct of 37), the confidence level (**α**) was set to 0.10, and the critical t-value is t_0.10, 28_ = 1.31. The calculated t_ci_, and t_cs_ of the three assays are 2.8 and 1.7 for cmEPO, 2.7 and 2.1 for hEPO, and 1.6 and 3.0 for CMV. All of these t-values are greater than the critical t-value of 1.31, demonstrating that the Ct from a target DNA and the Ct from an equal amount of corresponding ITC template in each duplex assay are statistically equivalent.

### Assay Precision

As shown in **Table 2**, the reproducibility of the assays was evaluated. Coefficients of variation (CV) from all six assays are within 3.2%. Furthermore, to determine the precision of the ITC^hEPO^ duplex assay, an additional experiment was conducted where eight replicates of the ITC^hEPO^ duplex assay were performed on three consecutive days using hEPO plasmid DNA in the presence of naïve human gDNA. The intra-assay CV is 1.3% and the inter-assay CV is 2.8%. These CVs of less than 3% demonstrate that the ITC^hEPO^ assay is highly reproducible. Moreover, one-way ANOVA was performed to determine the relationship between testing days and Ct from the individual hEPO and ITC^hEPO^ PCR reactions, and shows that each Ct does not vary significantly over the testing days (data not shown).

### Evaluation of assay interference

**Figure 3:**
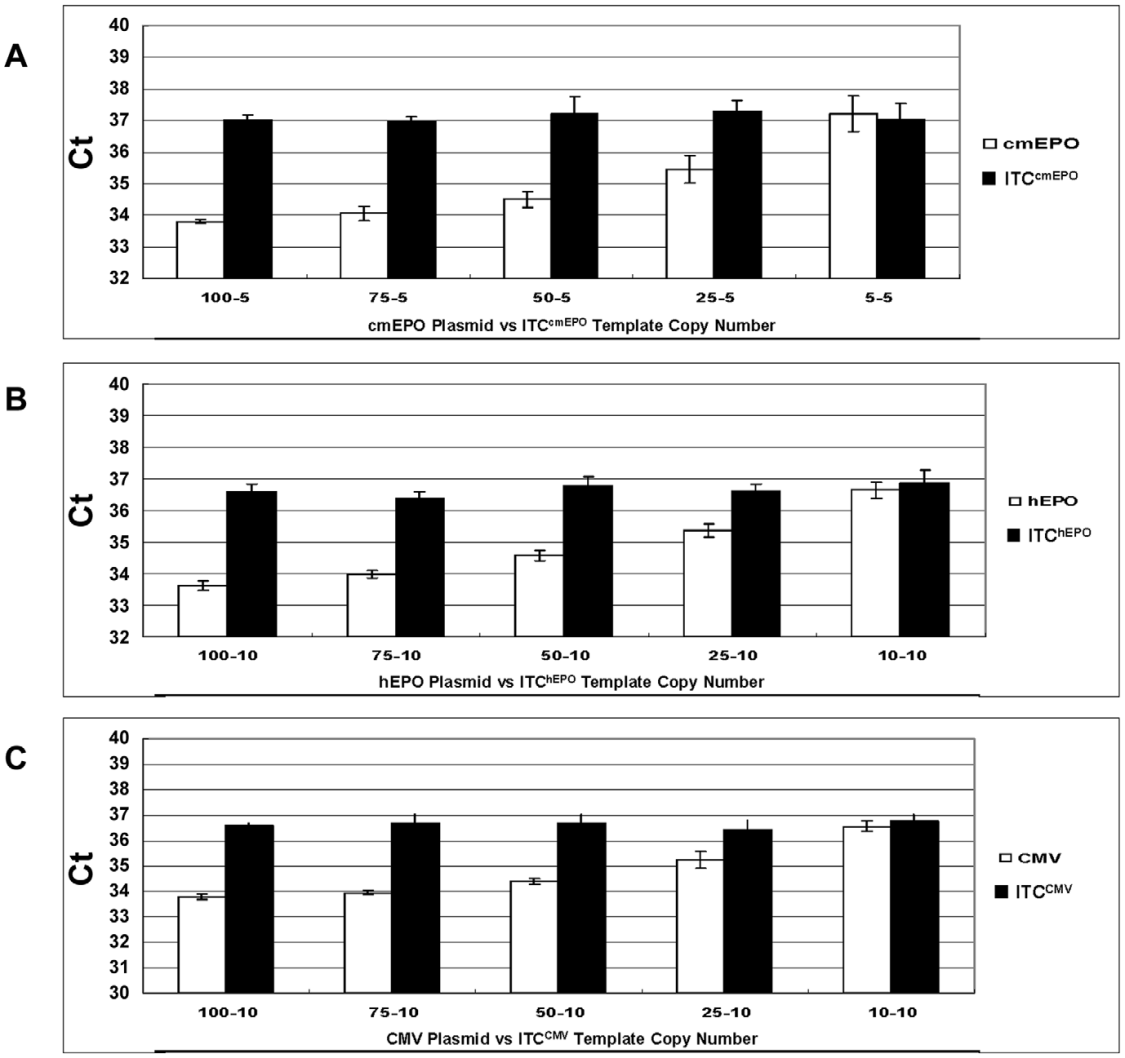
ITC duplex assay competition testing. The copy number of the ITC template was held at 5 copies (cmEPO ITC) or 10 copies (hEPO or CMV ITC) in each reaction, while the target template was titrated from 5 or 10 copies to 100 copies. Each reaction was repeated 5 times in the presence of 500 ng naïve gDNA to obtain the mean Ct value. **Panel (A)** ITC^cmEPO^ duplex assay, **Panel (B)** ITC^hEPO^ duplex assay and **Panel (C)** ITC^CMV^ duplex assay.

The target probe and its corresponding ITC probe are designed to detect two unique DNA sequences in one reaction. However, the two amplification systems share the same pair of primers, thus, possible competition between the target PCR reaction and the ITC PCR reaction was analyzed for each of the three ITC duplex assays. Experiments were performed where the copy number of the ITC template was held at 5 copies (cmEPO ITC) or 10 copies (hEPO or CMV ITC) in each reaction, while the target template was titrated from 5 or 10 copies to 100 copies, which is at the upper range of rAAV copies seen in 500 ng of macaque WBC gDNA at late timepoints following intramuscular injection [32]. As shown in **Figure 3**, the observed Ct from the ITC probe is stable while the Ct from the corresponding target probe increases according to the decrease in target copy number in the presence of 500 ng naïve gDNA. All three assays show the same pattern, which demonstrates that the target template does not interfere with the ITC detection in this copy number range. In the cases where the target copy number is over 100 copies, then an unequivocal signal will be detected by the target probe, even if an ITC signal is not detected, and will warrant further analysis of the sample.

**Figure 4:**
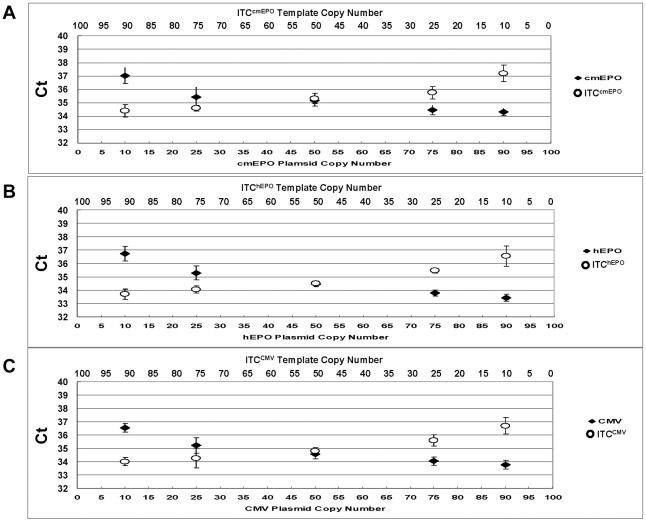
ITC duplex assay interference testing. Target plasmid (♦) and ITC template (O) were amplified in one reaction in the presence of 500 ng naïve gDNA to test the influence on the Ct between the two DNA targets. The total copy number of the plasmid and ITC template was maintained at 100. The target plasmid copy number is 10, 25, 50, 75, 90 and the ITC template copy number is 90, 75, 50, 25, 10 from left to right. Each reaction was repeated 5 times to obtain the mean Ct value. **Panel (A)** ITC^cmEPO^ duplex assay **Panel (B)** ITC^hEPO^ duplex assay and **Panel (C)** ITC^CMV^ duplex assay.

In addition, testing was performed to evaluate the dynamic range of all the three ITC duplex assays (**Figure 4**). The total number of the two templates was maintained at 100 copies. For each duplex assay, a plasmid harboring the target was titrated reciprocally with the ITC template in the presence of 500 ng naïve gDNA. The Ct of both probes illustrates that the Ct values for each template changed only with the template amount, without interference from the other template. As a result, if no signal is obtained from the ITC template it is most likely due to an inhibitor present in the sample, and the test will be invalid and require further analysis.

### Transduced Macaque blood sample testing

The performance of the ITC assay format was evaluated on the WBC DNA samples taken from macaques transduced *in vivo* with rAAV vectors and previously analyzed by traditional real-time PCR, where the actual copy number was determined at each timepoint [32]. The ITC^cmEPO^ duplex assay was conducted on both rAAV1 and rAAV8 *in vivo* samples. For the ITC testing, a 500 ng DNA sample and 5 copies of ITC^cmEPO^ template were amplified simultaneously in the presence of the cmEPO primers, the cmEPO probe, and the cmEPO ITC probe, and both fluorescence signals were recorded to obtain the Ct's. Each sample was tested repeatedly 5 times in order to acquire the mean and standard error for statistical analysis. Comparing the mean of cmEPO Ct and ITC^cmEpo^ Ct, the tested samples are defined to be positive (cmEPO Ct is less than or equal to the ITC^cmEpo^ Ct) or negative (cmEPO Ct is greater than ITC^cmEpo^ Ct with P-Value<0.05). As shown in **Table 4**, the ITC^cmEPO^ duplex assay results from both rAAV1 and rAAV8 injected animals are consistent with the previous absolute copy number data. Samples having more than two copies per 500 ng DNA are positive in the ITC duplex assay, meanwhile, the pre-injected samples test negative. The Student's t-test was applied and P-values of <0.001 were obtained when the absolute value of the difference between cmEPO cDNA and ITC^cmEPO^ template copy numbers is larger than five, demonstrating that this Ct difference is statistically significant. In addition, there is little to no competition of the target with the ITC, since the Ct's of the ITC for both animals at all vector copy numbers is ∼37 (CV = 1.5%). Furthermore, the samples do not appear to contain inhibitors since the Ct = 37 is similar to the Ct seen in TE (in the absence of gDNA, **Table 2**).

**Table 4:** ITC^cmEPO^ duplex assay testing of WBC samples from NHP transduced IM *in vivo* with rAAV1 or rAAV8.

Serotype/Time Point	Mean* cmEPO Ct (s.e.)	Mean ITC^cmEPO^ Ct** (s.e.)	Positive/Negative	Actual Copy Number***
rAAV1/Pre-injection.	40.00# (0)	37.00 (0.29)	Negative (P-value<0.0001)	0
rAAV1/3 Days p.i.	32.81 (0.10)	37.21 (0.15)	Positive (P-value<0.0001)	188
rAAV1/7 Days p.i.	35.92 (0.27)	37.48 (0.29)	Positive (P-value = 0.0019)	13
rAAV1/14 Days p.i.	36.94 (0.51)	37.46 (0.40)	Positive (P-value = 0.4350)	8
rAAV1/10 Weeks p.i.	38.49 (0.49)	37.67 (0.13)	Positive (P-value = 0.1066)	2
rAAV1/16 Weeks p.i.	38.32 (0.72)	37.16 (0.73)	Positive (P-value = 0.2913)	3
rAAV1/23 Weeks p.i.	39.91 (0.09)	37.18 (0.23)	Negative (P-value<0.0010)	0
rAAV8/Pre-injection.	40.00# (0)	37.43 (0.24)	Negative (P-value<0.0001)	0
rAAV8/3 Days p.i.	35.70 (0.49)	36.67 (0.12)	Positive (P-value<0.0875)	10
rAAV8/7 Days p.i.	36.68 (0.35)	36.99 (0.44)	Positive (P-value = 0.5923)	7
rAAV8/14 Days p.i.	37.44 (0.43)	37.43 (0.14)	Positive (P-value = 0.9795)	8
rAAV8/10 Weeks p.i.	40.00 (0)	36.82 (0.33)	Negative (P-value<0.0001)	0
rAAV8/16 Weeks p.i.	38.02 (0.68)	36.83 (0.68)	Positive (P-value = 0.2528)	2
rAAV8/23 Weeks p.i.	38.17 (0.54)	37.30 (0.19)	Positive (P-value = 0.1432)	3

* From five replicates of each 500 ng *in vivo* sample. s.e. = standard error, s.e., (in parenthesis).

** From an input of five copies of ITC^cmEPO^ template.

*** The actual copy number is from our previous study [32].

# The number of cycles of the real-time PCR program was set to 40.

### Testing of human WBC gDNA spiked with plasmid DNA

The plasmid pShuttle-CAG-hEPO-pA harboring the hEPO cDNA was spiked into 500 ng naïve human gDNA at the same copy numbers detected in the rAAV1 injected macaque shown in **Table 4**, and ten copies of ITC^hEPO^ template was added to each reaction. **Table 5** shows that spiking with 188, 13 and 8 copies of pShuttle-CAG-hEPO-pA were designated as positive, meanwhile 0 and 2 copies were designated negative when compared to the ITC^hEPO^ Ct.

**Table 5:** ITC^hEPO^ duplex assay testing.

Copy Number of Spiked Plasmid	Mean* hEPO Ct (s.e.)	Mean ITC^hEPO^ Ct** (s.e.)	Positive/Negative
0	40.00# (0)	36.93 (0.42)	Negative (P-value<0.0001)
188	32.60 (0.44)	36.48 (0.33)	Positive (P-value<0.0001)
13	36.63 (0.28)	37.12 (0.27)	Positive (P-value = 0.2426)
8	36.88 (0.28)	36.84 (0.28)	Positive (P-value = 0.9172)
2	39.80 (0.20)	37.32 (0.37)	Negative (P-value<0.0001)

* From five replicates of pShuttle-CAG-hEPO-pA plasmid DNA spiked into 500 ng naïve human gDNA. Standard error, s.e., (in parenthesis).

** From an input of 10 copies of ITC^hEPO^ template.

# The number of cycles of the real-time PCR program was set to 40.

The ITC duplex assay format is also capable of being used to detect human infectious agents such as viruses or bacteria that have sequences that are non-homologous to human genomic DNA. Cytomegalovirus causes many human infections [43], and the viral load in blood is very important for clinicians to evaluate patients' prognosis. As a proof of concept, The CMV immediate early promoter in plasmid pSSV9-MD2-cmEPO was used as a PCR target for two reasons. The first is to compare the sensitivity of the EPO intron-spanning PCR to a target that has no competition with human genomic DNA, and the second is to demonstrate the applicability to using the ITC approach for infectious disease diagnosis and treatment monitoring. As can be seen in **Tables 1** **and** **2**, the CMV and hEPO assays had similar 10 copy sensitivities.

For the ITC^hEPO^ and ITC^CMV^ duplex assays, we determined the difference in the Ct's between the target sequence and corresponding ITC template at the 95% confidence interval. For this purpose, the ITC templates were held constant at 10 copies and a titration of 5, 10 and 20 copies of target plasmid were added to evaluate the ability to determine positive (10 and 20 copies) from negative (5 copies) samples. Four different naïve human gDNA samples spiked with 5, 10 and 20 copies of hEPO or CMV target plasmid were amplified in the presence of 10 copies of their corresponding ITC template. The Ct's were analyzed by Student's t-test and One-Way ANOVA. **Tables 6** **and** **7** show that, with all four human gDNA sources, the Ct from 5 copies of hEPO or CMV target sequence is significantly larger than 10 copies of corresponding ITC template, the Ct from 20 copies is significantly smaller than 10 copies of ITC template, and no statistical difference (evaluated by equivalence testing – data not shown) was seen when the target and ITC copy numbers were equal at 10 copies each. Two-Way ANOVA analysis was performed and showed that the copy number detected was independent of the different gDNA samples (data not shown).

**Table 6:** Human EPO cDNA detection in the presence of different naïve human gDNA samples.

Human gDNA	5∶10*			10∶10**			20∶10***		
	Mean§ hEPO Ct	Mean ITC^hEPO^ Ct	P-Value	Mean§ hEPO Ct	Mean ITC^hEPO^ Ct	P-Value	Mean§ hEPO Ct	Mean ITC^hEPO^ Ct	P-Value
**1**	37.36 (0.84)	36.25 (0.53)	0.0368	36.80 (0.41)	36.70 (0.83)	0.8853	35.36 (0.46)	36.95 (0.50)	0.0482
**2**	38.03 (0.40)	36.25 (0.24)	0.0052	36.88 (0.32)	36.99 (0.58)	0.8663	35.17 (0.39)	36.41 (0.14)	0.0168
**3**	37.45 (0.85)	36.39 (0.38)	0.0253	36.94 (0.44)	37.14 (0.83)	0.7840	35.18 (0.44)	37.46 (0.59)	0.0143
**4**	37.62 (0.33)	36.24 (0.10)	0.0040	36.91 (0.35)	37.00 (0.79)	0.8924	35.34(0.45)	36.97 (0.50)	0.0425

§ From five replicates of pShuttle-CAG-hEPO-pA plasmid DNA spiked into 500 ng naïve human gDNA. Standard error (in parenthesis).

* 5 copies of plasmid and 10 copies of ITC template.

** 10 copies of plasmid and 10 copies of ITC template.

*** 20 copies of plasmid and 10 copies of ITC template.

The number of cycles of the real-time PCR program was set to 40.

**Table 7:** CMV promoter detection in the presence of different naïve human gDNA samples.

Human gDNA	5∶10*			10∶10**			20∶10***		
	Mean† CMV Ct	Mean ITC^CMV^ Ct	P-Value	Mean† CMV Ct	Mean ITC^CMV^ Ct	P-Value	Mean† CMV Ct	Mean ITC^CMV^ Ct	P-Value
**1**	38.88 (0.35)	36.27 (0.08)	<0.0001	36.77 (0.08)	36.62 (0.12)	0.3259	36.21 (0.09)	36.65 (0.11)	0.0172
**2**	38.37 (0.32)	36.70 (0.12)	0.0011	36.56 (0.14)	36.97 (0.15)	0.3333	36.11 (0.11)	36.69 (0.08)	0.0025
**3**	38.55 (0.42)	36.82 (0.13)	0.0045	36.78 (0.07)	36.74 (0.13)	0.7807	36.23 (0.14)	36.91 (0.13)	0.0081
**4**	38.83 (0.32)	36.73 (0.18)	0.0005	36.67 (0.12)	36.67 (0.08)	0.9355	36.06 (0.23)	36.71 (0.04)	0.0183

† From five replicates of pSSV9-MD2-cmEPO plasmid DNA spiked into 500 ng naïve human gDNA. Standard error (in parenthesis).

* 5 copies of plasmid and 10 copies of ITC template.

** 10 copies of plasmid and 10 copies of ITC template.

*** 20 copies of plasmid and 10 copies of ITC template.

The number of cycles of the real-time PCR program was set to 40.

## Discussion

We report developing a real-time PCR assay format for detecting homologous and non-homologous exogenous DNA. These tests are useful for infectious disease diagnosis, gene therapy clinical trial safety, and gene doping surveillance where the control of false negative and false positive results, and the assurance of true positive results is required. These procedures facilitate the procurement, preparation, and testing of samples to detect exogenous DNA sequences in a user-friendly format. Furthermore, the ITC assay format described here is ideal for clinical testing labs since 1) it is a “single tube" assay [sample DNA+master mix (2 primers, 2 probes and ITC template)], 2) it is specific and sensitive with samples internally controlled, 3) it is fast: 2 to 3 hours (including set up and analysis), 4) it requires no standard curve titration tubes (no external standards), 5) it is high throughput, capable of analyzing many samples at once, 6) it is automated: data captured by PCR machine and results can be transferred to centralized database(s), 7) no additional manipulations are needed for analysis such as gel electrophoresis, and 8) it reduces risk of laboratory contamination (a source of false positives) since no positive control plasmid is needed and uracil-N-glycosylase (UNG) prevents the re-amplification of carryover PCR products in subsequent analyses.

When designing and validating an ITC duplex assay, in addition to considering GC content, primer and probe Tm, and amplicon length, additionally, lack of cross complementary of the primers and two probes, the targets or amplicons, or any combination of these is needed [44]. Moreover, since both assays are amplified in a single tube, amplification competes for the same dNTPs and polymerase. One more challenge in the detection of cDNAs in the presence of gDNA is that the assay must detect the exon-exon junction of the cDNA, which greatly restricts the choice of primers and probes.

The individual assays described here have a sensitivity of 10 copies or better in the presence of 1.5E5 cellular genome copies and maintain linearity over 8 logs. The two probes in each duplex assay give similar Ct values when detecting the same amount of their respective sequences, and this was supported statistically using equivalence testing. Interference and competition testing has shown that target probe, ITC probe, target template and ITC template will not inhibit each other in the range of 10–100 copies. Furthermore, similar amplification of the PCR targets was achieved in individual PCR reactions compared with the duplex PCR reactions.

The maintenance of rAAV sequences in WBC of nonhuman primates [32] and humans [25] provides an easily accessible target for the surveillance of gene doping. Our previous experiments in the nonhuman primate were designed to test the feasibility of detecting vector sequences in blood long-term following IM injection and to gain insight into testing humans. We have designed the ITC duplex PCR format described in this paper with the expectation that athlete samples will be sent to testing laboratories for preparation and PCR analysis, and then the test results will then be sent to central databases for analysis and trending. The methods developed to detect EPO also provide the basis to detect other prohibited gene doping targets.

Previous studies have introduced internal controls using cellular housekeeping genes to control for possible PCR inhibitory factors, but they were not used to set a copy number threshold of the exogenous sequence target [45]–[46]. Moreover, competitive PCR methods are used to quantify DNA copy number, however, this approach is limited by the necessity of assembling multiple competitive reactions for a single determination and, most notably, the need for a post-PCR detection and analysis step [47]. On the other hand, each ITC duplex assay detects two different DNA sequences in one reaction by real-time PCR, with the Ct from the ITC probe being a threshold to evaluate if the samples are positive or negative. The ITC^cmEPO^ duplex assay testing on blood samples from macaques transduced *in vivo* with rAAV vectors shows consistent results with our previous quantitative data [32]. Furthermore, to simulate the testing of human samples, the ITC templates were held constant and the corresponding hEPO or CMV target plasmid was titrated in the presence of naïve human genomic DNA to evaluate the ability to determine positive from negative samples.

ITC assays can be used to detect heterologous sequences (infectious agents) or exogenously added homologous cDNA sequences (gene transfer vectors). Thus, ITC assays can be applied to pre-clinical animal biodustribution studies and legitimate human gene therapy clinical trials to determine the presence or absence of gene transfer vector sequences in different tissues, where the ITC can control for different types of inhibitors potentially present in different tissue samples. Likewise, molecular tests for infectious disease diagnosis, prognosis, and evaluation of response to therapy could benefit from an ITC assay approach. The ITC assay format is also applicable to gene doping surveillance testing as a means to deter the illegitimate use of gene transfer vectors for athletic performance. Compared to traditional real-time PCR, the ITC assay format has advantages for detecting exogenous DNA.
